# Women in the Singlet Fission World: Pearls in a Semi-Open Shell

**DOI:** 10.3390/molecules26102922

**Published:** 2021-05-14

**Authors:** Joanna Stoycheva, Julia Romanova, Alia Tadjer

**Affiliations:** Faculty of Chemistry and Pharmacy, Sofia University, 1164 Sofia, Bulgaria

**Keywords:** excited states, photovoltaics, solar cells, singlet/triplet excitons, diradical character, SF mechanism

## Abstract

Singlet fission, a multiple exciton generation process, can revolutionize existing solar cell technologies. Offering the possibility to double photocurrent, the process has become a focal point for physicists, chemists, software developers, and engineers. The following review is dedicated to the female investigators, predominantly theorists, who have contributed to the field of singlet fission. We highlight their most significant advances in the subject, from deciphering the mechanism of the process to designing coveted singlet fission materials.

## 1. Introduction

A Chinese proverb says that “Pearls don’t lie on the seashore. If you want one, you must dive for it.” There are such female divers, amas, in the realm of singlet fission. 

According to symbolism, buried within the shell, the pearl from the Chinese proverb represents “hidden knowledge and is highly feminine” [1]. Though women are excellent research partners and always work in mixed scientific teams, in accordance with the topic of the Special Issue, Women in Physical Chemistry, this review honors women who have dived in the singlet fission deep-sea and revisits their co-authored works on the subject with a focus on the theoretical studies. As representatives of a female group, we would also like to share our strategy for the design of singlet fission materials. 

Singlet fission is a truly special phenomenon, the study of which we can compare to swimming underwater—captivated and amazed by the depths, while being lured to mysterious and still unknown events. In the world’s green race to utilize the naturally generated energy, singlet fission is a milestone in solar-to-chemical energy conversion. 

## 2. Singlet Fission Renaissance

All natural and artificial systems that are able to transform solar energy—from leaves on a tree to photovoltaic cells—do so through really fast processes at the atomic and molecular levels. While nature can elegantly control these processes, synthetic light-harvesting is tricky because of the low yields of photovoltaic cells. For the most successful inorganic photovoltaic cells, the maximum efficiency is only 33% [2]. While inorganic solar cells are broadly available on the market, conventional organic photovoltaic devices are far from the large-scale manufacturing stage due to their much lower and non-competitive efficiency. Fortunately, it became clear recently that singlet fission in organic materials is a promising way to circumvent the 33% limit [3].

**Figure 1:**
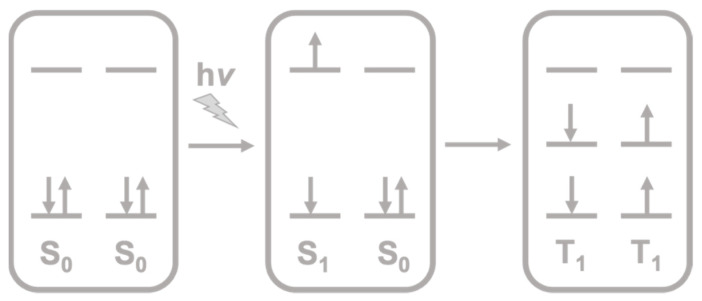
Schematic representation of the singlet fission process.

Singlet fission is a process that occurs in organic molecules upon the absorption of light. It produces a singlet excited state (S_1_) in one molecule that is shared with a neighboring one in the ground state (S_0_), thus splitting into two triplet excited states (T_1_) (Figure 1). 

Initially, the two triplets are coupled in an overall singlet biexciton (^1^TT). The process is thus spin-allowed and, consequently, proceeds rapidly. The existence of this intermediate state has been a subject of a long-standing controversy [4]. Jenny Clark et al. just recently resolved the latter, providing the first direct spectroscopic evidence that ^1^TT is a real stage in the fission of the singlet state [5]. The next step is the dissociation of the biexciton into two independent triplet excitons. If harnessed in photovoltaic cells, singlet fission could boost sizably the efficiency of the latter—the doubled number of created excitons could lead to a doubled number of free charges.

**Figure 2:**
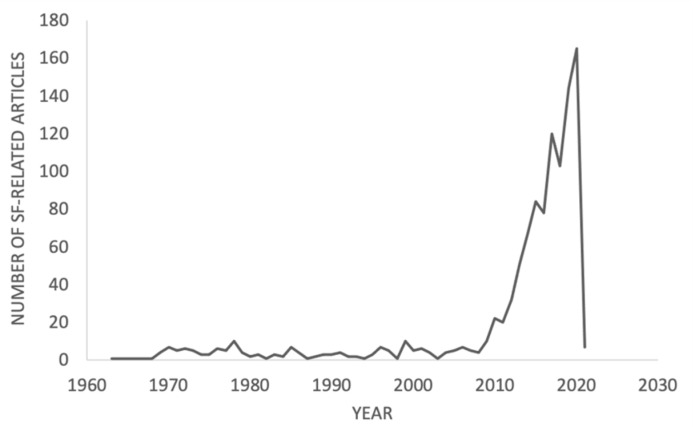
The number of singlet-fission(SF)-related articles published from 1963 until 2021.

The most emblematic publication on singlet fission, cited more than a thousand times to date, was the review authored by Millicent Smith and Josef Michl in 2010 [6]. M. Smith and her mentor defined the minimum energy conditions for a material to exhibit singlet fission. The process is thermodynamically efficient when it is exoergic or isoergic, which means that the excitation energy to the first singlet excited state exceeds the doubled excitation energy to the first triplet excited state: E(S_1_) ≥ 2E(T_1_). To ensure that singlet fission outruns the competing processes, there is another condition concerning the excitation energy to the first and second triplet excited states: E(T_2_) ≥ 2E(T_1_). M. Smith and J. Michl summarized the theory behind the process and listed the singlet fission materials examined until then. Their seminal work turned out to be the fuel needed, so the next three years saw enormous research interest in singlet fission materials that could contribute to the reduction of the environmental burden. In 2013, the two authors published their second updated review on the topic, recapitulating the newly gained knowledge [7]. Since then, an avalanche of reviews followed [8,9,10,11,12,13,14,15,16,17,18,19,20,21,22,23,24], and the process of singlet fission has emerged as the frontrunner in the field of organic photovoltaics, attracting the keen interest of more and more researchers from all over the world (Figure 2).

## 3. The Open Shells

The feasibility conditions derived by M. Smith and J. Michl comprise a good estimate at the molecular level whether a given chromophore is suitable for singlet fission application. Recently, an alternative descriptor, related to the propensity of a chromophore for singlet fission, was found—the diradical character (DRC) of the molecule [25]. This is possible because DRC, as well as the first energy condition, depends on the frontier molecular orbitals. DRC is an experimentally non-measurable property that quantifies how “open” is the electronic shell of the system and presents a measure of its stability and chemical reactivity. It takes a value ranging from 0 (closed shell) to 1 (pure diradical). Proceeding from the former to the latter case, one certainly comes across the diradicaloid situation where the magic of singlet fission happens. It has been demonstrated that molecules with a semi-open-shell character, namely with a low-to-intermediate diradical character, turn out to be successful singlet fission candidates. 

Right here comes one of the bottlenecks in the singlet fission quest: the potential chromophores with semi-open-shell character cannot be reliably treated by conventional single-reference quantum-chemical methods. The precise theoretical description of diradicals’ wave functions requires at least two configurations. 

One of the broadly-used, inexpensive, single-reference methods for examining electronic excitations in molecules is TD-DFT [26]. However, when used as an instrument for the search of diradicals as singlet fission materials [27], it often gives false positive results, as shown by Jin Wen and co-workers [28], and the explanation comes to light when compared to higher-level computations. Benchmark TD-DFT calculations showed that the problem of the method is a triplet instability [29]—some triplet excitation energies become significantly too low [30]. Considering that singlet fission produces two electronic triplet states, the stability of the latter is of paramount importance.

One of the main contributors to the development of the new, computationally cheap, and accurate treatment of singlet fission chromophores is Anna Krylov. She proved that the multi-configurational nature of open-shell singlet states could be represented with the help of a single-reference formalism—just one spin flip is required [31]. In the so-called spin-flip method, these states can be accessed by single excitations that flip the spin (α→β) on one of the electrons. Depending on the way that one accounts for the electron correlation, one can obtain varying degrees of accuracy [32]. Still, the method is unconstrained by system-dependent parametrizations and can be applied to much larger systems than the conventional multi-reference methods [33]. The spin-flip algorithm is encoded and implemented in the Q-Chem package [34].

## 4. Mechanism, Rate, and the Supramolecular View

The mechanism is definitely among the most intriguing problems in the singlet fission field, and female scientists have their fingerprint in its investigation.

The feasibility conditions and the diradical character quantify the singlet fission propensity of a material at the molecular level, but they represent only one piece of the puzzle of the phenomenon. A wider picture of the singlet fission process can be obtained by taking the interaction between the chromophores (chromophore coupling) into account. It has been demonstrated that the intermolecular interaction has a crucial impact on the final performance [35] and that singlet fission efficiency is governed by the type and strength of chromophore coupling. 

Electronic configurations, which can describe the singlet fission process in a pair of chromophores, A and B, are the excitonic (EX: S_0_(A)S_1_(B) and S_1_(A)S_0_(B)), charge-resonance (CR: linear combination of A^+^B^−^ and A^−^B^+^), and multi-excitonic (^1^ME: two triplets coupled into a singlet: T_1_(A)T_1_(B)) [36]. Charge-resonance character, however, is present with a small weight in the EX and ME wave functions, although it has been neglected for a long time by researchers. A. Krylov showed that the presence of CR in EX and ME is extremely important for the states’ coupling and, in some cases, is the only key to capturing it [37].

The two most frequently considered mechanisms of the singlet fission process are the direct nonadiabatic transition from S_1_ to ^1^ME and the transition mediated by charge transfer. Usually, for the description of nonadiabatic processes, one employs diabatization procedures. A. Krylov and co-workers, however, introduced an alternative approach based on adiabatic wave functions, considering the norm of the reduced one-particle transition density matrix. The model was constructed in order to explain the experimentally observed trends in the rate of singlet fission in a series of three acenes: tetracene, pentacene, and hexacene [38]. However, it was also used to explain some more puzzling cases. We already emphasized how important the interaction between the two chromophores is, especially in solids. Speaking of crystal packing, we have to refer to the compound 1,3-diphenylisobenzofuran, which was proven to be an efficient singlet fission material in 2010 [39]. However, four years later, it turned out that this is true only for one of its crystal forms. In fact, there is an impressive difference in the triplet yield of two structurally similar modifications of the material—200% vs only 10% [40]. Thus, the natural question is: what makes the one modification favorable and the other one unfavorable? There are two factors explaining how an excited state evolves from a singlet to two triplets [41]. On the one hand is the energetics—singlet fission is about 0.02 eV less endothermic in the successful form. On the other hand is the coupling. The form of 1,3-diphenylisobenzofuran, in which the process is faster, is characterized by a stronger coupling due to the efficient mixing of CR configurations into the S_1_ to ^1^ME states [37]. As already said, the group of A. Krylov was the first to notice the importance of the charge resonance character. The theoretical method of her group holds promise because its results were in good agreement with experimental data [40]. According to the latest discoveries, however, dimer-based models of singlet fission are not sufficient, prompting the need for oligomer-based ones. Via a detailed comparison between calculated and experimentally measured data, evidence emerged that a general model should consider at least three molecules simultaneously in order to reproduce the real system behavior [42]. The example of 1,3-diphenylisobenzofuran is only one of many that serves as proof of how important molecular packing is for the singlet fission efficiency [43,44].

**Figure 3:**
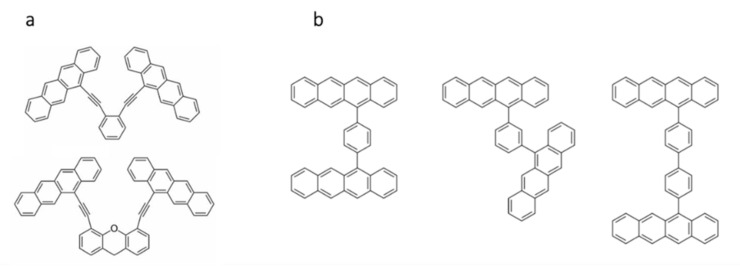
Staggered (**a**) and coplanar (**b**) configurations of tetracene moieties examined by A. Krylov et al. [46]. Reprinted with permission from [46], copyright 2016 Royal Society of Chemistry.

Another interesting illustration of the importance of chromophore coupling was presented and studied by the group of A. Krylov in 2016, with tetracene playing the leading role. Dimers with coplanar and staggered arrangements of tetracene moieties, bridged by various conjugated linkers (Figure 3), were examined with the aim of finding theoretical explanation for an experimentally observed result [45,46,47]. The difference between the two structural types is the degree of overlap between the individual chromophores. Structures with staggered tetracene rings exhibit larger overlaps than those with non-stacked ones. In agreement with the expectations and experiments, the former show a faster singlet fission rate, relative to neat tetracene, while the latter exhibit a slower rate.

The lack of guidelines for packing that increases the singlet fission rates inspired an international research team to formulate some. A procedure that seeks the most optimal geometry of π-electron chromophore pairs was found [48]. The simplest example of a π-electron system is ethylene, so the choice of a pair of ethylenes as a model was practical. For simplification, the two molecules were considered as rigid bodies with six available degrees of freedom. Diving in the six-dimensional space of geometries, the authors found these mutual dispositions for which the rate of conversion of a singlet exciton to a singlet biexciton was the highest. The computer code was named “Simple” because it was based on a series of simplifications and approximations. It examined only two molecules at a time and ignored the many body interactions. The matrix elements of the interaction Hamiltonian were obtained using the frontier molecular orbitals model, where only two electrons from each chromophore were used for constructing an active space. All the remaining ones acted as a fixed frame. The resulting active space consisted of four electrons, two occupied and two unoccupied orbitals (FEFO), described in a basis set of natural atomic orbitals. Considering that extended Hückel theory (EHT) allows for the rapid assessment of the matrix elements for excitations fully localized on the individual molecules and for those delocalized between them, the EHT approximations [49] were also applied. 

It is important to underline that the aim of the researchers was not to obtain the exact numerical values but to rank the geometries with respect to efficiency. Since close results for the matrix elements were obtained with the far more accurate ab initio methods (CASSCF for the ground state and ROHF for the excited states), the procedure could be classified as trustworthy. Though the latter was developed for the simple model system of ethylene pairs, it could be applied to larger and even non-planar π-electron systems. 

Ria Broer is amongst the explorers of another successful application of this strategy to a pair of cibalackrot molecules [50]. Cibalackrot is an indigo derivative dye showing promising photovoltaic properties, even if not gainful for singlet fission. Nevertheless, it is believed that smart modifications could result in successful materials. That is why the authors aimed to position the two molecules in a way that produced rapid singlet fission. The geometries provided by “Simple” were checked against ab initio results. Though the procedure relies on a series of approximations, it was found to be in good agreement with high-level theory. The recipe of the authors, however, still needs improvements—their method considers only two molecules at a time and does not account for competing processes.

The most recent success of R. Broer and co-workers in the singlet fission world is the joint project GronOR [51], named after the working places of the project partners: University of Groningen and Oak Ridge National Laboratory. GronOR is a non-orthogonal configuration interaction (NOCI) [52] code “for describing the electronic structure of molecular assemblies in terms of individual molecular wave functions or molecular fragment wave functions” [53]. It finds its application in the process of singlet fission in terms of calculating effective electronic state couplings. 

Not only theorists are involved in the investigation of singlet fission mechanism details. The feasibility conditions may be considered sufficient for the conversion of a singlet exciton into a pair of coupled triplet ones, but the dissociation of the latter is another bottleneck for the efficient utilization of the process. Nathalie Pace and co-workers conducted a non-trivial experiment that showed the difference between singlet and triplet excitons in the most studied singlet fission system: pentacene [54]. Being a good electron donor, pentacene needs an electron-accepting partner, and 12 different molecules were tested as such in the study. It turned out that triplet dissociation is five orders of magnitude slower than singlet dissociation. A possible explanation of this finding, suggested by the authors, is grounded on the different nature of the two types of excitons—the triplet ones are more localized, thus limiting the electronic overlap between the donor and the acceptor [55].

## 5. From Molecular Design to Lab Realization

Understanding the mechanism of singlet fission is definitely one of the main challenges in the field, but, going back to the molecular level, there are also plenty of open questions to the scientific community. A relatively small number of known singlet fission chromophores exists, and the structural variety among them is modest. This is a clear indication that the knowledge of the structure–property relationship in such systems is still limited and that we are on the eve of a molecular design revolution in the field. The discovery of new singlet fission chromophores will increase the structural diversity, which, in turn, will broaden the avenues for fine-tuning of the molecular response at the supramolecular level.

The article that coined the term “Singlet Fission” was published in 1963 after an extensive study on anthracene crystals [56]. Anthracene, being the smallest member of the acene family with three conjugated benzene rings, is in fact a fruitful base for modeling of singlet fission materials. The process dynamics in the next representatives of the acene series—tetracene and pentacene—were examined by a research team including J. Clark. It turned out that singlet fission in tetracene is a relatively slow process with dynamics, independent of temperature, and the singlets of pentacene fission to pairs of triplets really fast, independent of the excitation wavelength [57,58,59]. The fact that singlet fission in tetracene does not require thermal activation and the demonstration that the process is both rapid and efficient in pentacene are the reasons why these materials were the principle ones in historical studies of singlet fission chromophores.

Nandini Ananth et al. showed that the pattern of bonding is critical for the absorption characteristics of polyacene dimers. Using a shrewd combination of theoretical methods and experimental certifications, they created a simple rule that allowed for the construction of dimers, intensely absorbing in the visible range, while the monomers have an essentially dark S_0_–S_1_ transition. Based on the type of the monomer orbital interaction, predictions can be made regarding whether such a long-wavelength absorption could emerge, borrowing intensity from a bright short-wavelength transition [60].

As was demonstrated [61], one of the promising design strategies is based on doping polycyclic aromatic hydrocarbons (PAHs). The latter obey the 4n+2 Hückel rule [62], but a doping that changes the π-electrons by 2 makes the systems antiaromatic (4n π-electrons) and, at the same time, raises their singlet fission proclivity.

Inspired by the antiaromaticity idea, we recently estimated theoretically the potential of boron doping in PAHs for the creation of new singlet fission chromophores. Our work was focused on 14 isoelectronic diboron-doped anthracene and phenanthrene derivatives [63]. While the acenes have already been on the singlet fission stage for almost six decades, to our knowledge, this is the first study to also address phenanthrenes. The series of compounds was specially constructed in order to reveal the intimate relationship between molecular topology, open-shell character, and singlet fission propensity. Since singlet fission proclivity starts with suitable excitation energies in isolated molecules, the chromophores were modeled by using the high-level RASSCF//RASPT2 method—a protocol known to provide accurate results for excitations in good agreement with experimental spectral data if used correctly [64].

**Figure 4:**
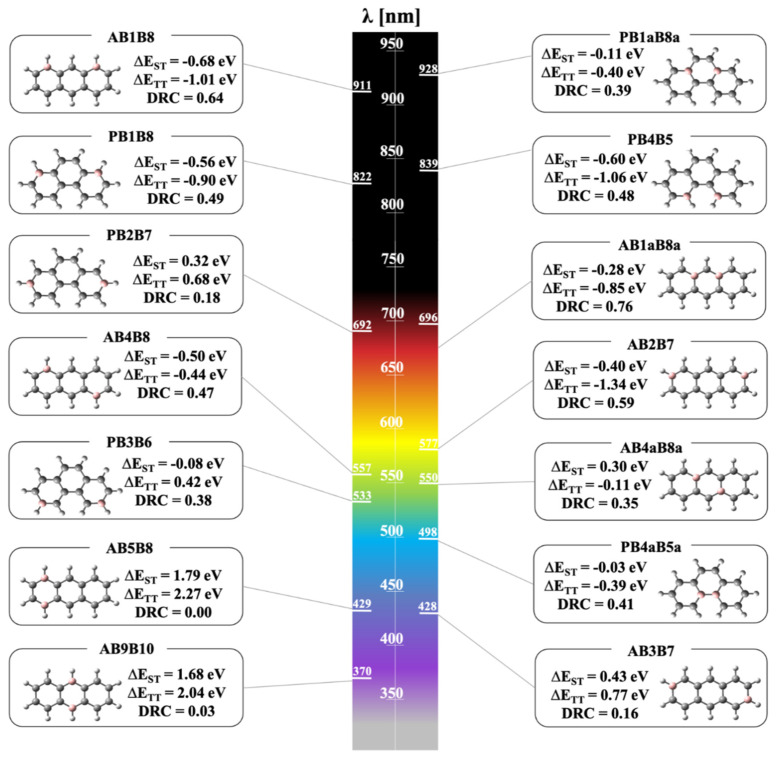
Molecular set investigated by J. Stoycheva et al. [63]. The compounds’ names contain the following labels: A (anthracene derivative), P (phenanthrene derivative), and B (boron), as well as numbers that show the position of the heteroatoms in accordance with the IUPAC numbering of the pristine hydrocarbons. The wavelength, λ (nm), of the first allowed for electronic transition (f ≥ 0.02), diradical character (DRC), ΔE_ST_ (eV) = 2E(T_1_) − E(S_1_), and ΔE_TT_ (eV) = 2E(T_1_) − E(T_1_). Reprinted with permission from [63]. Copyright 2020 American Chemical Society.

Our results clearly demonstrated the power of boron doping and topological approach in the design of new singlet fission molecules based on PAHs. We observed that, depending on the doping position, boron substitution can drastically modulate the optical properties of the compounds and can even turn small-sized PAHs into efficient singlet fission materials. Moreover, our results demonstrated that boron atoms break the π-conjugation in the PAHs, outlining hydrocarbon substructures with high singlet fission potential and suggesting boron-doped graphene and nanotubes as new potential materials in the field. The open-shell character and stability of such hydrocarbon substructures were explained by the spin-polarization mechanism and resonance structures, which allows for the design of new singlet fission chromophores even without the use of computational power. On the basis of their photophysical properties, we proposed four new potential chromophores that satisfy the two energy criteria for efficient singlet fission and are likely to absorb photons with reasonable oscillator strengths in the visible range while featuring low-to-intermediate DRC (Figure 4). A benefit of our molecules is their relatively small size: nowadays, special attention is being paid to small chromophores—they are easily modeled computationally and concomitantly imply a high exciton density by weight and volume. The only shortcoming of the aforementioned boron-doped materials is their stability, which is believed to be relatively low, as a consequence of their open-shell character. As our singlet fission library needs to be expanded in order to address practical issues, we suggested the chemical functionalization of the compounds as a stabilizing tactic.

The recipe of J. Wen et al. for the design of singlet fission chromophores is: “take a perfect biradical and perturb it covalently in the direction of producing an ordinary closed-shell ground state molecule, not too little and not too much” [28]. The natural products of this technique are captodative biradicaloids—open-shell species carrying both an acceptor and a donor group that are stabilized by direct interaction between their radical centers. The authors demonstrated that the presence of donor–acceptor groups gives rise to a charge-transfer character of the excited states and proposed the solvent as an additional parameter for the fine-tuning of the singlet fission propensity. 

An alternative recipe was promoted in a paper co-authored by N. Ananth [27]: take a stable small molecule and perturb it both too little and too much by the introduction of captodative doping through the simultaneous replacement of any two carbons by a boron and a nitrogen or other heteroatomic combinations with varying topology. After the strategy with tuning DRC with appropriate substituents was applied, several useful design rules were formulated and three prospective singlet fission candidates were suggested.

A good practice for tuning the diradical character of carbene-based materials was recommended by Achini Japahuge et al. in 2019 [65]. Utilizing spin-flip mixed-reference formalism, the authors investigated a series of cyclic (alkyl)(amino)carbene (CAAC) dimers, differing in the electronegativity of the α-C substituent and the type and length of the linker. It was found that higher substituent electronegativity stabilizes LUMO of the monomers, thus increasing the DRC and satisfying the feasibility conditions. The linkers were either C_sp_ (C_2_ and C_4_) or C_sp2_ (p-phenylene). The C_sp_ bridges increased DRC with elongation, but they invoked substantial structural changes upon excitation that obstructed singlet fission. On the other hand, structural modifications were negligible for the C_sp2_ spacer. Thus, the interplay between substituent electronegativity and linker type was pointed out as molecular design guideline for avoiding significant structural reorganizations upon excitation [66,67]. 

Most of the studies on singlet fission have been focused on intermolecular singlet fission, in which the coupling of the two chromophores occurs through space. The dependence on the connection between the two separate units, the difficult experimental control, the need for optimal molecular packing, and ingenious crystal engineering are only some of the predicaments to be outwitted before we can freely control this process. 

Using a pentacene dimer model and an elegant diabatization scheme, N. Ananth et al. managed to analyze the singlet fission mechanism, proving that CR and CT structures mediate the single-to-multiexcitonic through-space transition, which is sensitive to intermolecular distance and orientation and which can be tuned by crystal compression in a specific direction [68].

**Figure 5:**
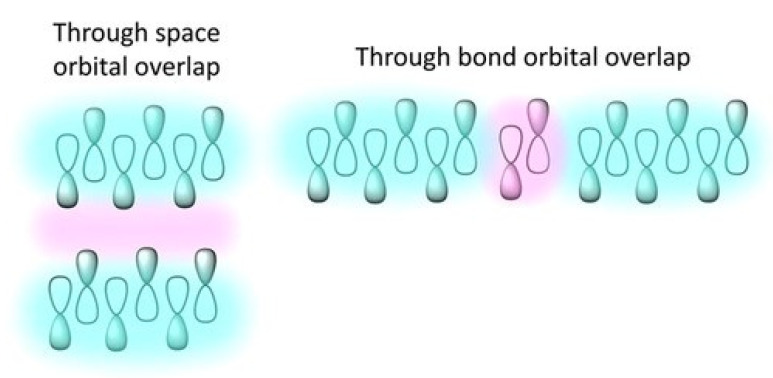
Orbital overlap of chromophores through space and through bonds. Reproduced with the permission from [73], copyright 2020 AIP Publishing.

Singlet exciton, however, can also interact with its neighboring ground state molecule through bond to create a triplet pair. In other words, the two chromophores can be covalently bound in a single molecule [69,70]—which is the definition of intramolecular SF (iSF). In the latter, the two triplet excitons are “located” on different parts of the same molecule. The presence of a chemical fragment, connecting the two centers, allows for an interaction through the bond, which is impossible in molecular crystals (Figure 5). Theoretical calculations have shown that the interactions through covalent bonds are stronger than those through space [46].

In combined computational and experimental studies on the covalently bound dimers of pentacene, the group of N. Ananth demonstrated that the direct coupling iSF mechanism is operative and mediated by vibronic coupling to intramolecular modes of the covalent dimer, thus avoiding the CT step [71]. The direct mechanism proved to predict correctly the variation of iSF rate with the twist angle between the monomers and to be independent of solvent polarity. A number of spacers was tested [72], providing different lengths, rigidities of separation, and types of conjugation between the monomers, thus revealing that homoconjugated dimers display better the wanted excited-state dynamics with sizably reduced recombination rates compared to conjugated dimers with comparable rates of singlet fission.

**Figure 6:**
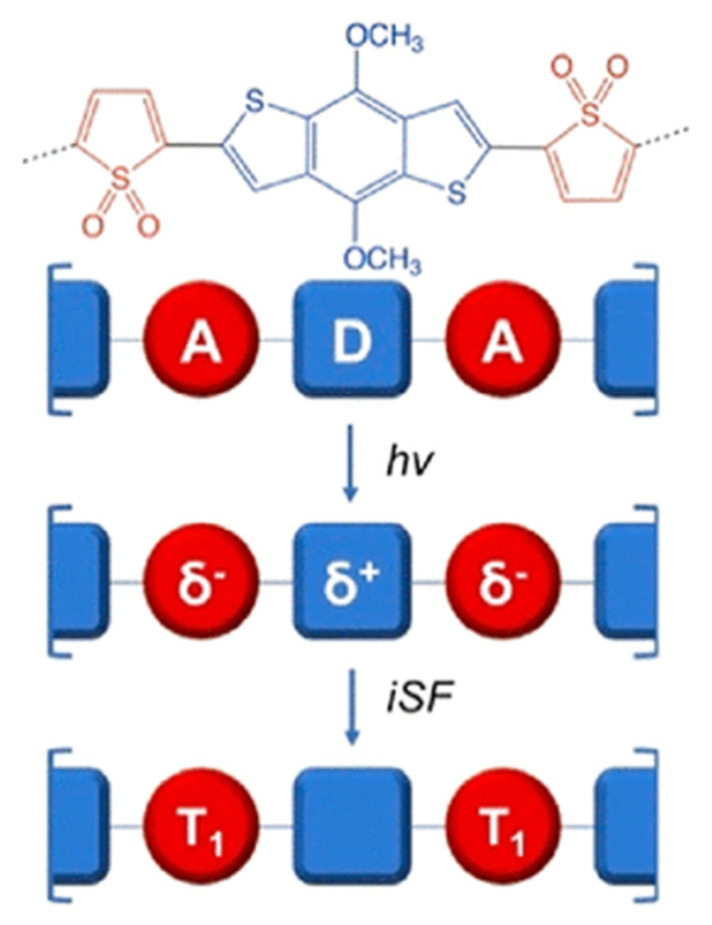
The process of iSF in donor–acceptor copolymers. Reproduced with a permission from [75], copyright 2020 American Chemical Society.

Another powerful advantage of chemically bound singlet fission fragments is that the process becomes an intrinsic molecular property. In other words, the iSF system comes “pre-assembled” and no longer needs adjustment to a partner [73]. Conjugated polymers are examples of such pre-assembled systems. Among them of recent research interest are the donor–acceptor (D–A) copolymers [74]. There, the photon absorption center is the donor core, which transfers its charge to the acceptor moiety. Elucidating the iSF mechanism in D–A copolymers, represents an even more interesting intellectual challenge because the process requires the inspection of one more molecular unit. Following the A–D–A polymeric chain, it is important to consider one donor and two acceptor units (Figure 6). 

The lowest singlet excited state S_1_ displays a charge transfer character from the donor to the acceptor unit(s), and the other relevant states, the triplet ones, are localized on the acceptors [75]. In this way, the latter are separated by the donor unit, which is believed to suppress the recombination of triplets. 

The good charge transport properties and tunable structures have made donor–acceptor copolymers the materials of choice for Clémence Corminboeuf in her theoretical investigations. She is the woman who took the task to guide the way towards providing a cost-effective computational protocol “for an accelerated screening of promising iSF donor–acceptor pairs” on her shoulders while minimizing the number of computations [76]. In a very recent study, she and her co-workers built a library of 81 dimers of donor–acceptor copolymers, and this was only their first step towards the creation of a database of such dimers for engineering iSF copolymers. The library consists of well-established heterocycles that can be easily synthesized. 

The researchers chose to correlate the charge transfer character of S_1_ with the frontier molecular orbitals of the constituent donor and acceptor units. In a D–A copolymer, the donor center is characterized by a low ionization potential wherefrom the HOMO of the copolymer originates. Correspondingly, the acceptor part of the molecule defines the copolymer LUMO [77]. Thus, the obtained material will have a narrower HOMO–LUMO gap than the band gaps of the constituent parts. The group of C. Corminboeuf revealed how, depending on the ordering of the frontier molecular orbitals, the excitation from the donor to the acceptor can compete with local excitations. Additionally, an expression that reveals the relationship between the excited state character of donor–acceptor dimers and their frontier orbitals energies was proposed. In the age of artificial intelligence and robotics, one of the most promising strategies for molecular design is the high-throughput screening of massive databases. Such a database of donor–acceptor polymer pairs and molecular linkers permits the performance prediction of a certain material in a photovoltaic device and can incredibly accelerate the process of discovering new and better iSF materials [76]. 

**Figure 7:**
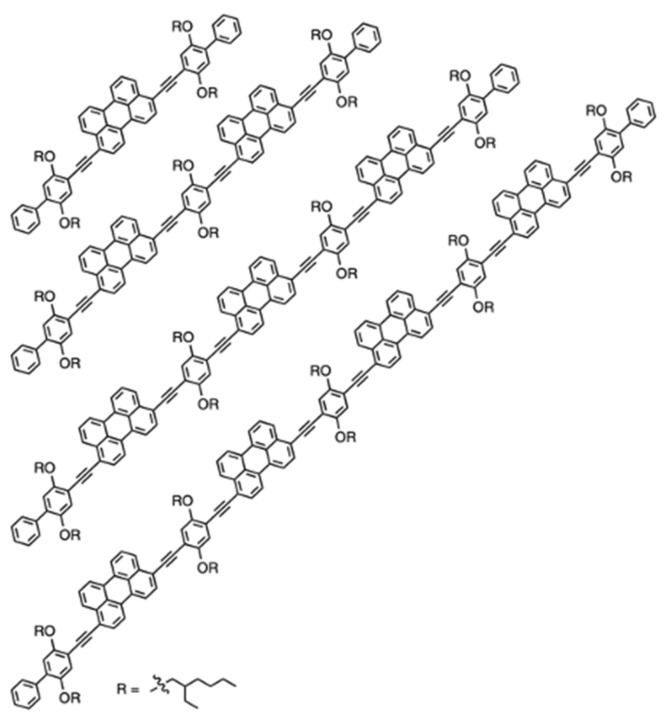
Oligomer structures consisting of one, two, three, and four perylene units, as examined by N. Korovina et al. [78]. Reprinted with a permission from [78], copyright 2020 Nature Research.

The investigation of the still-enigmatic instances of endothermic singlet fission also needs at least three coupled units, as shown by Nadezhda Korovina and co-workers [78]. In their study, they chose perylene as base chromophore. Due to N. Korovina’s rich successful experience with analogous multi-chromophoric structures [79], the right linker was quickly selected to be 1,4-dialkynyl-2,5-bis(ethylhexyloxy)-benzene, and oligomers were built in a head-to-tail fashion. In order to evaluate what influence the number of perylene units would have on singlet fission propensity, the authors created a series of oligomer structures (Figure 7). The used linker is known for its ability to introduce strong electronic coupling between chromophores, which is believed to lead to a fast conversion between the excitonic and multi-excitonic states. In fact, the study concluded that dimers cannot sustain well-separated triplets and, hence, effective triplet yield. Trimer, tetramer, and longer oligomers do not suffer from spatial hindrance and can therefore support stable multi-excitonic states. The efforts of N. Korovina and her colleagues in understanding endothermic singlet fission were crowned with a patent registration in 2020 [80], with her being the leading author. 

Maria-Elisabeth Michel-Beyerle is another pioneer in the field. In 1978, her group reported excitation spectra of singlet fission into a pair of triplet excitons in organic crystals of anthracene and perylene [81,82,83]. Perylene is a photostable material with high absorption coefficients that exists in two forms. β-perylene crystals are monomeric and therefore similar to anthracene ones. α-perylene crystals are dimeric, which allows for the formation of excimers. The authors found the blue shift of the fission threshold in α-perylene crystals and explained this effect with the competition between singlet fission and excimer formation. More recent papers on the singlet fission phenomenon with the participation of M.E. Michel-Beyerle also appeared in 2012–2014 [84,85,86,87]. Unprecedented in the literature, G.G. Gurzadyan et al. observed direct singlet fission from two-photon excited upper electronic states in a single crystal of rubrene [84,85] and α-perylene [86]. A fast singlet fission process was found to proceed in both of them in three different ways: (i) from the upper excited electronic states S_n_, (ii) from the upper vibrational states of S_1_, (iii) and via two-step two-photon absorption to S_n_ [87].

Singlet fission offers routes to entirely new device architectures. In order for the latter to be technologically competitive, the precise control of energetics is required. There has been significant recent progress towards incorporating singlet fission layers into a heterojunction with a second light harvester and a lower bandgap. Such a hybrid device was produced in 2018 by combining crystalline silicon with tetracene interlayers [88]. It turned out stable, with c-Si remaining well-passivated by the organic film. This architecture is one of many that have the opportunity to open new horizons for the photovoltaics market [89,90].

## 6. Closing Words

Pearls are hard to find—before the discovery of the “cultured pearls” production, the pearl hunters used to find no more than three-to-four good pearls within a ton of mollusks. Understanding the details of the origin and nature of singlet fission will make this process “cultured.” The study of singlet fission is a challenge from both fundamental and applied points of view, and there are more and more female researchers who are giving their time and energy to unveiling the mysteries associated with the phenomenon. Some try to decipher its mechanism and others are application-oriented, aspiring the design and synthesis of novel singlet fission materials. Moreover, women venture to apply modern approaches, such as artificial intelligence, for designing singlet fission chromophores. In this respect, we would also like to mention our recently started project [91], devoted to the application of machine learning algorithms for the hunting of new singlet fission materials. 

We hope that this paper will motivate more women to start their scientific careers, opening the shells of knowledge, finding hidden pearls, and increasing the productivity of the physical chemistry community. 

## Data Availability

Data sharing is not applicable to this article because no new data were created or analyzed in this study.

## Sample Availability

Not available.
